# Prospective cohort study of corneal endothelial cell loss after Baerveldt glaucoma implantation

**DOI:** 10.1371/journal.pone.0201342

**Published:** 2018-07-26

**Authors:** Kentaro Iwasaki, Shogo Arimura, Yuji Takihara, Yoshihiro Takamura, Masaru Inatani

**Affiliations:** Department of Ophthalmology, Faculty of Medical Sciences, University of Fukui, Fukui, Japan; Xiamen University, CHINA

## Abstract

**Purpose:**

To evaluate corneal endothelial cell loss after Baerveldt glaucoma implantation.

**Methods:**

We prospectively evaluated changes in endothelial cell density (ECD) in the cornea of 59 patients (59 eyes) who underwent Baerveldt glaucoma implantation. Tubes were inserted into the anterior chamber in 45 eyes and pars plana in 14 eyes. The primary outcome measure was the change in corneal ECD after Baerveldt glaucoma implantation. Secondary outcome measures included relationships between corneal ECD and optical coherence tomography images of tube parameters (tube–cornea distance and tube–cornea angle) and prognostic factors for corneal ECD loss.

**Results:**

Corneal ECD decreased significantly by 9.2% at 12 months after surgery (P = 0.001). In the anterior chamber Baerveldt glaucoma implantation group, corneal ECD at the tube insertion quadrant decreased significantly by 9.6% at 3 months (P = <0.001), 10.7% at 6 months (P = <0.001), and 13.1% at 12 months (P = <0.001). Corneal ECD at the center decreased significantly at 6 months after surgery (7.2% loss; P = 0.012) and at 12 months after surgery (12.1% loss; P = 0.001). Corneal ECD at the contralateral quadrant decreased significantly at 12 months after surgery only (10.3% loss; P = 0.004). In the pars plana Baerveldt glaucoma implantation group, no significant loss of corneal ECD was found in any corneal areas at any post-surgery follow-up visits. Tube-cornea angle was negatively correlated with the rate of corneal ECD loss at the tube insertion quadrant; r = −0.55 (P = 0.0013). In multivariable analyses, exfoliation glaucoma and narrower tube–cornea angle were significant prognostic factors for severe corneal ECD loss (P = 0.0068 and P = 0.046, respectively).

**Conclusions:**

Anterior chamber Baerveldt glaucoma implantation causes corneal endothelial cell loss. Corneal endothelial cell loss starts at the tube insertion quadrant. Exfoliation glaucoma and narrower tube-corneal angle are associated with severe loss of corneal endothelial cells.

## Introduction

Tube-shunt surgery using glaucoma drainage implants has become increasingly popular in refractory glaucoma in patients who have undergone one or more intraocular surgeries [1]. The Tube Versus Trabeculectomy Study showed that tube-shunt surgery using the Baerveldt glaucoma implant (BGI) of a 350-mm^2^ silicone endplate offers higher success rates and lower early postoperative complication rates in patients that have undergone prior trabeculectomy and/or cataract surgery than does trabeculectomy [2, 3]. The study showed that persistent corneal edema was present in 16% of patients with BGI and 9% of those with trabeculectomy [3].

Corneal endothelial cell loss is a serious but frequent complication after tube-shunt surgery [4–6]. A quantitative follow-up analysis of corneal endothelial cell density (ECD) was conducted to compare between tube-shunt surgery using the Ahmed glaucoma valve and trabeculectomy, and this study demonstrated significantly greater reduction of corneal ECD in tube-shunt surgery than in trabeculectomy [7]. Insertion of the tube in the anterior chamber is believed to be involved in the loss of ECD [4, 8–10] because corneal endothelial cell loss after tube insertion into the pars plana is mild and comparable to that observed after simple cataract surgery [11]. In fact, pars plana insertion of the Ahmed glaucoma valve shows less ECD reduction than does anterior chamber insertion [12]. ECD reduction also depends on tube position in the anterior chamber. ECD of the corneal area near the tube insertion point decreases more than that of other areas [8, 9, 13–15]. Recent studies using anterior segment-optical coherence tomography (AS-OCT) show that a short distance between the tube and cornea endothelium is associated with severe loss of ECD [15]. The purpose of the present study was to identify the relationship between corneal ECD loss and tube position after BGI using a 350-mm^2^ endplate.

## Materials and methods

### Patient selection

This study was approved by the Institutional Review Board of Fukui University Hospital, Fukui, Japan. This study was registered with the University Hospital Medical Information Network Clinical Trials Registry of Japan (identifier University Hospital Medical Information Network: UMIN 000007812; date of access and registration: April 24, 2012). The protocol followed the guidelines of the Declaration of Helsinki. Written informed consent was obtained from all patients after providing a detailed explanation of the procedures involved.

This prospective clinical cohort study evaluated corneal ECD reduction after BGI of a 350-mm^2^ endplate (BG101-350 or BG102-350; Abbott Medical Optics, Abbott Park, IL, USA). Patients were recruited between April 1, 2012 and March 31, 2015 at Fukui University Hospital using the following inclusion criteria: age of 20 years or older and having refractory glaucoma, such as secondary glaucoma, neovascular glaucoma, or glaucomas involving a wide conjunctival scar after previous failed filtration surgery or retinal surgery. Exclusion criteria were eyes planned for primary surgery using the BGI, eyes with a history of tube-shunt surgery, and eyes with congenital glaucoma.

### Surgical procedures

One surgeon (MI) performed all surgeries. The surgical procedure was as follows. In brief, all tube-shunt surgeries were performed using a BG101-350 or BG102-350. The silicone tube was completely occluded with an 8–0 absorbable vicryl suture (Coated VICRYL; Ethicon, Somerville, OH, USA) to minimize the risk of early postoperative hypotony. A fornix-based conjunctival flap was created after administration of subconjunctival and sub-Tenon’s xylocaine anesthesia. The silicone plate was preferentially placed in the superotemporal scleral quadrant. If the superotemporal quadrant had intensive surgical conjunctival scarring, the plate was placed in one of the other quadrants. After the endplate was placed under the rectus muscles, it was fixed on the scleral surface with a 7–0 nylon about 10 mm from the corneal limbus. A scleral tunnel was created in the anterior chamber with a 23-gauge needle to insert the tube into the anterior chamber. Tube insertion into the vitreous cavity through pars plana using a BG102-350 was performed in eyes that had undergone vitrectomy. As for the procedure for tube insertion into the vitreous cavity, a 20-gauge needle was used to penetrate the vitreous space. Then, the tube with the Hoffmann elbow (BG102-350) was transsclerally inserted into the vitreous space and sutured to the sclera with a 9–0 nylon. To reduce the frequency of early postoperative intraocular pressure (IOP) elevation, Sherwood slits were created in the tube with the needle of the 9–0 nylon. The tube was covered with a half-thickness rectangular scleral flap or a scleral patch graft supplied by the eye bank. The scleral patch and conjunctival flap were sutured using a 9–0 nylon and 8–0 absorbable vicryl.

All patients received similar postoperative topical medication, with 0.5% levofloxacin for 3 weeks and 0.1% betamethasone sodium phosphate for 6 months.

### Data collection

Patient data collected included sex, age, type of glaucoma, preoperative IOP, endplate position, corneal ECD, postoperative IOP, best corrected visual acuity (BCVA), number of glaucoma medications, number of previous intraocular surgeries, and any postoperative complications. The logarithm of the reciprocal of the decimal BCVA was used to approximate the logarithm of the minimal angle of resolution.

The initial study-related visit was scheduled 2 weeks after surgery; thereafter, visits were conducted 1, 3, 6, 9, and 12 months later. IOP, BCVA, and number of glaucoma medications were assessed before surgery and at all postoperative visits. Complications were also assessed at all postoperative visits.

### Corneal endothelium

The corneal endothelium was analyzed by a noncontact-type specular microscope (Konan Specular Microscope XI FA-3709P; Konan Medical Inc., Hyogo, Japan). All examinations were carried out at the central, superior, nasal, temporal, and inferior points of the cornea. One image at each point was taken to count corneal ECD. Patients were asked to look at the fixation light in the equipment. A manual center-dot method was used to measure the corneal ECD. The purpose of the study was masked to the examiner.

Corneal data were measured before surgery and at 3, 6, and 12 month postoperative visits. We classified the five corneal points into three areas for analysis: tube insertion quadrant, center, and contralateral quadrant. For example, when the endplate was placed in the superotemporal quadrant, the tube insertion quadrant was calculated as the average of the superior and temporal points, and the contralateral quadrant was calculated as the average of the nasal and inferior points.

### Tube–cornea distance and tube–cornea angle

The tube in the anterior chamber was measured with AS-OCT (SS-1000, CASIA, Tomey, Aichi, Japan). Anterior segment scans were acquired at an angle parallel to the tube in the anterior chamber. Patients were asked to look at the fixation light in the equipment and not to move the eyeball during examination.

**Fig 1:**
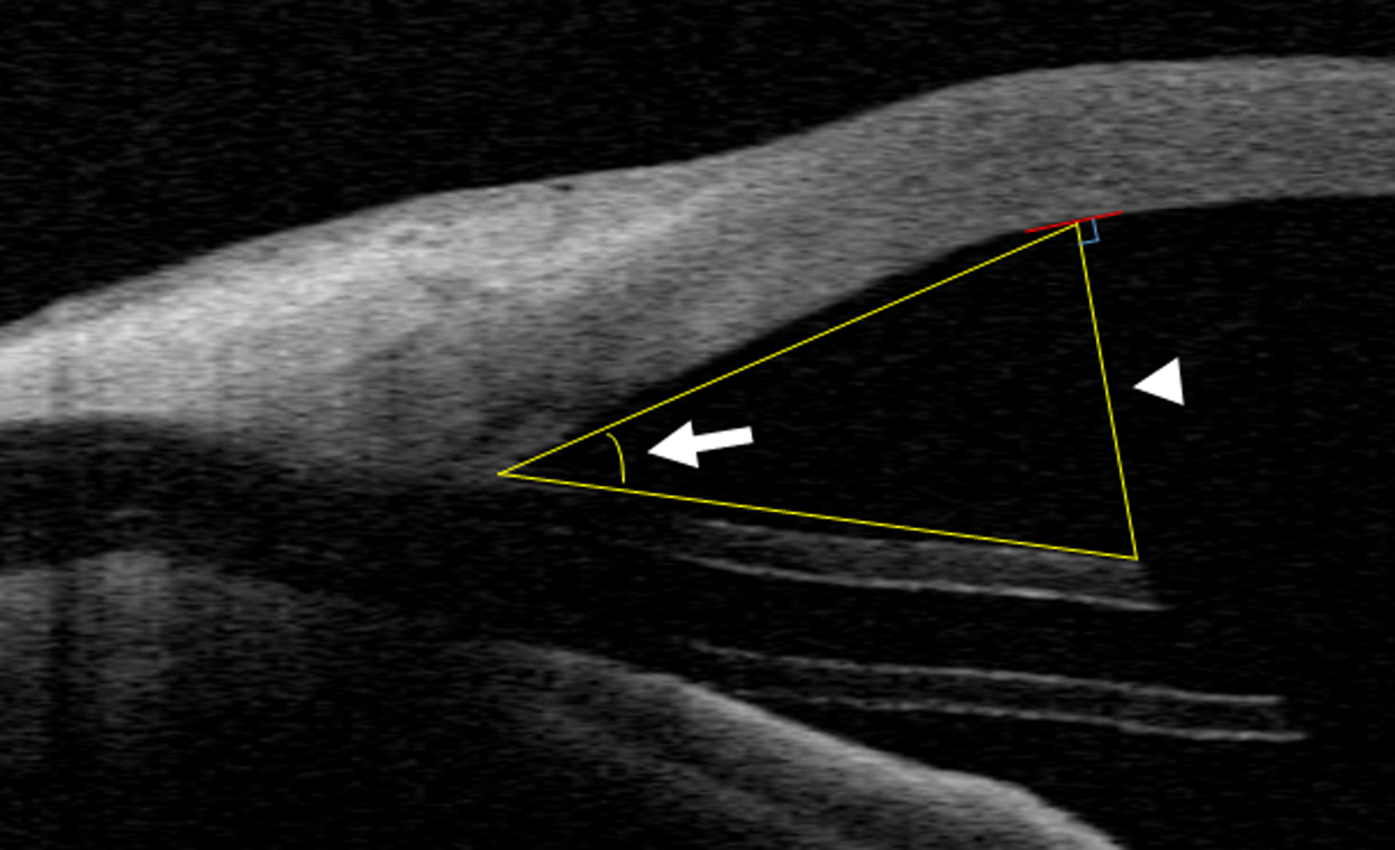
AS-OCT image of eyes with anterior chamber BGI. AS-OCT shows the tube of the Baerveldt glaucoma implant (BGI), the tube–cornea angle (arrow), and the tube–cornea distance (arrowhead).

Tube image was measured with AS-OCT at 1 month post-surgery. If a clear image of the tube was not obtained at 1 month due to corneal opacity after surgery, the tube image was taken at the next follow-up visit. On the tube image, the tube–cornea distance was measured by the perpendicular line from the superior tip of the tube to the corneal endothelium. A triangle was drawn using the tube insertion point in the anterior chamber angle and the perpendicular line. The tube–cornea angle was measured using the triangle (Fig 1). These data were analyzed by two independent observers (KI, SA) using the AS-OCT software.

### Outcome measures

The primary outcome measure was the reduction of corneal ECD after BGI. Secondary outcome measures included the relationships between corneal ECD and the OCT images of the tube parameters (tube–cornea distance and tube–cornea angle), IOP, number of glaucoma medications, and postoperative complications.

### Statistical analysis

Univariate comparisons between subgroups were performed using paired *t* test with Bonferroni correction, unpaired *t* test, chi–squared test, and Fisher’s exact test. The longitudinal repeated measures were analyzed using one-way repeated measures ANOVA and two-way repeated measures ANOVA. Correlations between corneal ECD and tube parameters were analyzed using the Spearman rank correlation coefficient. Multivariate analysis was performed to determine the prognostic factors for an ECD reduction of greater than 30% using a logistic regression model. *P*-values < 0.05 were considered statistically significant. Data were analyzed with the JMP statistical package, version 11.2.0 (SAS Institute, Inc. Cary, NC, USA).

## Results

### Patient characteristics

In total, 59 patients were enrolled in the study. Table 1 summarizes the patients’ baseline characteristics. In 45 patients (76%), a BG101-350 tube was inserted into the anterior chamber (acBGI group), whereas in the other 14 patients (24%), a BG102-350 tube was inserted into the pars plana (ppBGI group). In 44 of the 59 patients (75%), the endplate was placed in the superotemporal quadrant. Patients in the acBGI group were significantly older than those in the ppBGI group (P = 0.010). The significant difference was found in the type of glaucoma between the acBGI and ppBGI groups (P = <0.01). In 12 out of 14 ppBGI eyes (86%), the type of glaucoma was neovascular glaucoma. No other statistically significant differences in preoperative status were found between the 2 groups. In the acBGI group, follow-up of two patients ceased during the study period, as removal of the implant was performed due to plate escape, and another three eyes were lost to follow-up without any patient complications. Data before loss to follow-up were included in the analysis.

**Table 1:** Preoperative patient characteristics.

Characteristics	Total (n = 59)	acBGI (n = 45)	ppBGI (n = 14)	*P*-value
Age, mean ± SD, years	68.4 ± 12.3	70.7 ± 11.5	61.1 ± 12.6	0.010
Sex, n (%)				0.36
Men	30 (51%)	21 (47%)	9 (64%)	
Women	29 (49%)	24 (53%)	5 (36%)	
LogMAR BCVA	0.64 ± 0.6	0.62 ± 0.6	0.70 ± 0.6	0.65
Lens status, n (%)				0.36
Phakia	4 (7%)	4 (9%)	0 (0%)	
Pseudophakia	53 (90%)	40 (89%)	13 (93%)	
Aphakia	2 (3%)	1 (2%)	1 (7%)	
Type of glaucoma, n (%)				<0.01
Primary open-angle glaucoma	15 (25%)	15 (33%)	0 (0%)	
Exfoliation glaucoma	16 (28%)	15 (33%)	1 (7%)	
Neovascular glaucoma	15 (25%)	3 (7%)	12 (86%)	
Other secoundary glaucoma	13 (22%)	12 (27%)	1 (7%)	
Preoperative IOP, mean ± SD, mmHg	31.3 ± 10.0	30.4 ± 9.5	34.2 ± 11.0	0.23
Number of preoperative glaucoma medications, mean ± SD, n	3.3 ± 0.9	3.4 ± 0.8	3.1 ± 1.1	0.48
Number of previous intraocular surgeries, mean ± SD, n	2.4 ± 1.2	2.2 ± 0.9	3.0 ± 1.8	0.077
Endplate position, n (%)				0.10
Superotemporal	44 (75%)	30 (66%)	14 (100%)	
Inferotemporal	7 (12%)	7 (16%)	0 (0%)	
Superonasal	7 (12%)	7 (16%)	0 (0%)	
Inferonasal	1 (1%)	1 (2%)	0 (0%)	

acBGI = anterior chamber implantation of an Baerveldt glaucoma implant; ppBGI = pars plana implantation of an Baerveldt glaucoma implant; BCVA = best corrected visual acuity; IOP = intraocular pressure; logMAR = logarithm of minimum angle of resolution; SD = standard deviation. *P* values were calculated comparing between acBGI and ppBGI.

### Primary outcome measure

Forty-one patients completed the corneal ECD measurement at all the follow-up visits while 18 eyes could not complete corneal ECD measurement due to low photo quality. Corneal ECD (average of all five points) was 2,095 ± 605 cells/mm^2^ before surgery, and decreased significantly to 1,986 ± 582 cells/mm^2^ at 3 months after surgery (5.2% loss; P = 0.008), 1,961 ± 647 cells/mm^2^ at 6 months after surgery (6.4% loss; P = 0.001), and 1,903 ± 621 cells/mm^2^ at 12 months after surgery (9.2% loss; P = 0.001). In the tube insertion quadrant, corneal ECD decreased significantly at all postoperative follow-up visits. In the center and contralateral quadrant, corneal ECD decreased significantly at 6 months and 12 months after surgery. (Table 2).

**Table 2:** Change in corneal endothelial cell density in each area after Baerveldt glaucoma implantation.

(Cells/mm^2^)	Tube insertion (n = 41)	Center (n = 41)	Contralateral (n = 41)	Average
Preoperative	2,107 ± 625	2,109 ± 632	2,077 ± 605	2,095 ± 605
3 months	1,952 ± 584	2,046 ± 643	1,991 ± 577	1,986 ± 582
6 months	1,946 ± 683	1,982 ± 644	1,964 ± 631	1,961 ± 647
12 months	1,896 ± 656	1,906 ± 641	1,909 ± 604	1,903 ± 621
*P*-value				
All	<0.001^a^	<0.001^a^	0.001^a^	<0.001^a^
Pre vs 3 months	0.002^b^	0.554^b^	0.128^b^	0.008^b^
Pre vs 6 months	0.006^b^	0.010^b^	0.012^b^	0.001^b^
Pre vs 12 months	0.002^b^	0.001^b^	0.005^b^	0.001^b^

Data shown in mean ± standard deviation.

P-value

a, one-way repeated measures ANOVA

b, paired *t* test with Bonferroni correction

### Subgroup analyses of the primary outcome

We conducted subgroup analyses, dividing the 41 patients into two groups: (acBGI and ppBGI groups). In the acBGI group, corneal ECD at the tube insertion quadrant was 2,104 ± 639 cells/mm^2^ before surgery, and decreased significantly to 1,902 ± 543 cells/mm^2^ at 3 months after surgery (9.6% loss; P = <0.001), 1,879 ± 657 cells/mm^2^ at 6 months after surgery (10.7% loss; P = <0.001), and 1,828 ± 642 cells/mm^2^ at 12 months after surgery (13.1% loss; P = <0.001). Corneal ECD at the center decreased significantly at 6 months after surgery (7.2% loss; P = 0.012) and at 12 months after surgery (12.1% loss; P = 0.001). Corneal ECD at the contralateral quadrant decreased significantly at 12 months after surgery only (10.3% loss; P = 0.004) (Table 3). In the ppBGI group, no significant loss of corneal ECD was found in any corneal areas at any post-surgery follow-up visits (Table 4).

**Table 3:** Change in corneal endothelial cell density in each area after acBGI.

(Cells/mm^2^)	Tube insertion (n = 32)	Center (n = 32)	Contralateral (n = 32)
Preoperative	2,104 ± 639	2,076 ± 633	2,036 ± 605
3 months	1,902 ± 543	1,973 ± 586	1,947 ± 528
6 months	1,879 ± 657	1,927 ± 626	1,925 ± 614
12 months	1,828 ± 642	1,825 ± 609	1,826 ± 550
*P*-value			
All	<0.001^a^	<0.001^a^	<0.001^a^
Pre vs 3 months	<0.001^b^	0.122^b^	0.229^b^
Pre vs 6 months	<0.001^b^	0.012^b^	0.062^b^
Pre vs 12 months	<0.001^b^	0.001^b^	0.004^b^

Data shown in mean ± standard deviation; acBGI = anterior chamber implantation of an Baerveldt glaucoma implant

P-value

a, one-way repeated measures ANOVA

b, paired *t* test with Bonferroni correction

**Table 4:** Change in corneal endothelial cell density in each area after ppBGI.

(Cells/mm^2^)	Tube insertion (n = 9)	Center (n = 9)	Contralateral (n = 9)
Preoperative	2,119 ± 607	2,226 ± 650	2,223 ± 617
3 months	2,127 ± 721	2,308 ± 800	2,147 ± 739
6 months	2,185 ± 762	2,178 ± 704	2,105 ± 707
12 months	2,136 ± 685	2,194 ± 708	2,204 ± 725
*P-*value			
All	0.888^a^	0.450^a^	0.207^a^
Pre vs 3 months	>0.999^b^	>0.999^b^	>0.999^b^
Pre vs 6 months	>0.999^b^	>0.999^b^	0.146^b^
Pre vs 12 months	>0.999^b^	>0.999^b^	>0.999^b^

Data shown in mean ± standard deviation; ppBGI = pars plana implantation of an Baerveldt glaucoma implant

P-value

a, one-way repeated measures ANOVA

b, paired *t* test with Bonferroni correction

### Secondary outcome measures

#### Relationships between corneal ECD loss and tube parameters

In the acBGI group, the mean ± standard deviation of the tube–cornea distance was 1.7 ± 0.7 mm (range: 0.5 − 3.3 mm). There were no significant correlations between tube–cornea distance and the reduction rate of corneal ECD at 12 months after surgery in any areas. The mean ± standard deviation tube–cornea angle was 31.9 ± 9.6 degrees (range: 8.4 − 49.6 degree). The tube–cornea angle was negatively correlated with the reduction rate of corneal ECD at the tube insertion quadrant (r = −0.55, P = 0.0013) and center (r = −0.35, P = 0.047) (Table 5). There were no significant correlations between tube–cornea angle and the reduction rate of corneal ECD at the contralateral quadrant.

**Table 5:** Correlations between corneal ECD decrease rate and tube parameters.

	Corneal ECD decrease rate (%)					
	Tube insertion		Center		Contralateral	
n = 32	r	*P*-value	r	*P*-value	r	*P*-value
**Tube–cornea distance (mm)**	−0.12	0.50	−0.039	0.83	−0.35	0.051
**Tube angle (degree)**	−0.55	0.0013	−0.35	0.047	−0.16	0.40

ECD = endothelial cell density; *r*- and *P*-values indicate Spearman rank correlation coefficient and calculated probability, respectively

#### Comparison of IOPs, number of glaucoma medications, and complications between acBGI and ppBGI groups

Comparison of IOPs and number of glaucoma medications at follow-up visits between eyes in the acBGI and ppBGI groups (S1 Table) showed significantly higher postoperative IOP levels in the ppBGI group than in the acBGI group at 6 months (P = 0.010), 9 months (P = 0.003) and 12 months (P = 0.035) after surgery. The decrease over time of the number of postoperative glaucoma medications was significantly less in the ppBGI group than acBGI group (P = 0.049). However, no significant difference between the two groups was found in the number of postoperative glaucoma medications at any post-surgery follow-up visits.

As for postoperative complications (Table 6), vitreous hemorrhage occurred significantly more frequently in the ppBGI group (6 of 45 eyes; 42.9%) than in the acBGI group (0 eyes; P = < 0.01). Although shallow anterior chamber was observed in the acBGI group only (5 of 45 eyes; 11.1%), there was no significant difference between the two groups. No eyes encountered bullous keratopathy during the 12 months after surgery.

**Table 6:** Postoperative complications in acBGI and ppBGI eyes.

	acBGI (n = 45)	ppBGI (n = 14)	*P*-value
**Hyphema**	15 (33.3%)	5 (35.7%)	1.00
**Shallow anterior chamber**	5 (11.1%)	0 (0.0%)	0.33
**Choroidal detachment**	10 (22.2%)	2 (14.3%)	0.71
**Vitreous hemorrhage**	0 (0.0%)	6 (42.9%)	<0.01
**Retinal detachment**	1 (2.2%)	0 (0.0%)	1.00
**Tube occlusion**	0 (0.0%)	1 (7.1%)	0.24
**Implant infection**	1 (2.2%)	0 (0.0%)	1.00
**Escape of the plate**	2 (4.4%)	0 (0.0%)	1.00
**Hypotony**	1 (2.2%)	0 (0.0%)	1.00
**Bullous keratopathy**	0 (0.0%)	0 (0.0%)	NA

acBGI = anterior chamber implantation of an Baerveldt glaucoma implant; ppBGI = pars plana implantation of an Baerveldt glaucoma implant; NA = not applicable

### Determinants of severe corneal endothelial cell loss in acBGI

Patient characteristics, including age, type of glaucoma, tube–cornea angle, tube–cornea distance, number of preoperative glaucoma medications, preoperative IOP, and postoperative IOP, were evaluated as possible determinants of corneal ECD loss of greater than 30% from the preoperative corneal ECD at the tube insertion quadrant. In this study, 6 cases had > 30% corneal ECD loss from the preoperative corneal ECD at the tube insertion quadrant. Multivariate analyses using logistic regression models showed that exfoliation glaucoma and narrower tube–cornea angle were significantly associated with severe corneal ECD loss (P = 0.0068 and P = 0.046, respectively; Table 7).

**Table 7:** Multivariate analysis for determining prognostic factors for severe corneal endothelial cell loss at the tube insertion quadrant in acBGI using a logistic regression model.

Variable	RR (95% Cl)	*P*-value
Type of glaucoma (exfoliation glaucoma/other)	1630637.6 (1.89–3.34)	0.0068
Tube–cornea angle per degree	0.62 (0.09–0.99)	0.046
Tube–cornea distance per mm	39.3 (0.16–3.18)	0.30
Age per year	1.35 (0.89–4.32)	0.29
Preoperative glaucoma medication per each	37.1 (0.35–8.87)	0.19
Preoperative IOP per mmHg	1.05 (0.72–1.47)	0.78
Postoperative IOP per mmHg	0.28 (0.00–1.12)	0.11

95% CI = 95% Confidence Interval; IOP, intraocular pressure; RR, relative risk.

## Discussion

The primary outcome of the present study was the evaluation of corneal endothelial cell loss after BGI. Corneal ECD (average of all five corneal points) decreased significantly by 9.2% at 12 months after surgery. In the acBGI group, corneal ECD at the tube insertion quadrant decreased significantly by 9.6% at 3 months, 10.7% at 6 months, and 13.1% at 12 months. Corneal ECD at the center decreased significantly by 7.2% at 6 months, and 12.1% at 12 months. Corneal ECD at the contralateral quadrant decreased significantly by 10.3% at 12 months only. Corneal endothelial cell loss started at the tube insertion quadrant, then occurred in other areas. In the ppBGI group, no significant corneal ECD reduction was found in any corneal areas at any post-surgery follow-up visits. AS-OCT imaging for the tube in the acBGI group showed that, while no significant correlation was found between the tube–cornea distance and the reduction rate of corneal ECD, the tube–cornea angle was negatively correlated with the reduction rate of corneal ECD at the tube insertion quadrant and the center. Multivariable analyses confirmed that exfoliation glaucoma and narrow tube–cornea angle were significantly associated with severe corneal endothelial cell loss.

Four previous studies had evaluated changes in corneal ECD after tube-shunt surgery. Lee et al. [13] prospectively analyzed changes in corneal ECD of 41 eyes implanted with AGV in the anterior chamber. Koo et al. [14] retrospectively analyzed the relationship between AS-OCT of tube parameters and changes in corneal ECD of 39 eyes with AGV implanted in the anterior chamber. Seo et al. [12] retrospectively analyzed changes in corneal ECD of 18 eyes with AGV implanted in the anterior chamber and 18 eyes with AGV implanted in the pars plana. Tan et al. [15] prospectively analyzed the relationship between the AS-OCT of tube parameters and the changes in corneal ECD of 53 eyes with acBGI. The present study is unique because corneal ECD reduction and its determinants were prospectively evaluated in glaucoma eyes treated with the 350-mm^2^ endplate BGI including both acBGI and ppBGI.

Among the acBGI group, corneal ECD was significantly lower at the tube insertion quadrant during postoperative follow-up visits, which is consistent with the data shown in previous reports [13, 15]. Despite having the same endplate size as the acBGI group, no significant reduction of corneal ECD was observed postoperatively at the tube insertion quadrant in the ppBGI group. This discrepancy between the acBGI and ppBGI groups suggests that corneal ECD reduction should depend on the position of tube insertion rather than the position of the implanted endplate. The data also suggest that the distance between the corneal endothelium and the tube should be related to corneal ECD reduction. In addition to the corneal ECD reduction at the tube insertion quadrant in the acBGI group, corneal ECD was not significantly reduced at 3 months at the center, at 3 or 6 months at the contralateral quadrant, but was reduced at 12 months at the center and contralateral quadrants. The delayed loss at these areas might signify that loss of corneal endothelial cells near the tube in the anterior chamber caused a rebalance of corneal ECD in the entire cornea.

While previous studies showed that a shorter tube tip–cornea distance led to greater endothelial cell loss [14,15], the present analysis showed that corneal ECD reduction was significantly associated with tube–cornea angle, but not tube–cornea distance. One explanation for the inconsistency between our results and those of previous studies is that the longer tube–cornea distance (average = 1.7 mm) [14] in the present acBGI group might have resulted in a few eyes with shorter tube–cornea distances. Another explanation is that due to corneal curvature, a longer tube in the anterior chamber would have resulted in a longer tube–cornea distance even if the eye had a narrow tube–cornea angle. The multiple regression analysis shows that the tube–cornea distance depends on tube–cornea angle and tube length (angle; β = 0.86, P = <0.01, length; β = 0.87, P = <0.01: adjusted R^2^ = 0.91). Tube–cornea contact at the peripheral cornea in eyes with a narrow tube–cornea angle might have been the cause of the corneal endothelial cell loss at the tube insertion quadrant.

Exfoliation glaucoma includes corneal endothelial cell loss [16–18]. Inflammation in the anterior chamber and abnormal protein composition in the aqueous humor seem to be related to corneal ECD reduction in eyes with exfoliation [19]. Exfoliation is also a risk factor for corneal ECD reduction after lens extraction [20]. Inserting a tube in the anterior chamber of eyes with exfoliation might therefore enhance postoperative inflammation and protein accumulation in the aqueous humor.

The present study showed that the postoperative IOP was higher in the ppBGI group than the acBGI group. The rate of neovascular glaucoma was higher in the ppBGI group than the acBGI group in this study. The difference in type of glaucoma might have resulted in higher postoperative IOPs in the ppBGI group like a previous report [21]. Neovascular glaucoma is more refractory than other glaucoma. Our present study could not completely exclude the possibility that insufficient IOP reduction might results in less frequency of shallow anterior chamber and less loss of corneal ECD in the ppBGI group.

The present study had some limitations. Firstly, we did not collect certain clinical data during follow-up visits. Tube insertion in the anterior chamber causes iris damage and peripheral anterior synechia [4, 8, 19, 22, 23]. These complications may be associated with postoperative inflammation, which results in corneal ECD loss. Therefore, gonioscopic examination and the measurement of flare value by flare cell meter should have been included in our postoperative analyses. Secondly, corneal ECD might not have been measured at exactly identical points of the cornea across patients at all visits, although the same examiner did measure corneal ECD in each patient for consistency.

In conclusion, tube insertion in the anterior chamber during the BGI surgical procedure reduces corneal endothelial cells postoperatively, beginning with the loss in the corneal area around the tube insertion point. Exfoliation and narrower tube–cornea angle enhance corneal endothelial cells in acBGI.

## Supporting information

S1 Table Intraocular pressure and medical therapy at preoperative and follow-up visits. (DOCX)
