# Supplementary material for: Prospective cohort study of corneal endothelial cell loss after Baerveldt glaucoma implantation
Source: PLoS One. 2018 Jul 26;13(7):e0201342. doi: 10.1371/journal.pone.0201342 (PMC6062117; doi:10.1371/journal.pone.0201342)
Supplement: S1 Table — (DOCX) [file pone.0201342.s001.docx]

|  | **acBGI (n = 41)** | **ppBGI (n = 13)** | ***P*-value** |
| --- | --- | --- | --- |
| **IOP (mm Hg)** |  |  | 0.302^a^ |
| **Preoperative** | 30.0 ± 8.9 | 34.8 ± 11.2 | 0.112^b^ |
| **1 month** | 18.9 ± 7.4 | 18.4 ± 11.6 | 0.850^b^ |
| **3 months** | 15.4 ± 5.0 | 18.6 ± 7.2 | 0.075^b^ |
| **6 months** | 15.0 ± 4.9 | 19.7 ± 6.9 | 0.010^b^ |
| **9 months** | 13.4 ± 3.9 | 17.8 ± 5.4 | 0.003^b^ |
| **12 months** | 13.4 ± 3.9 | 16.2 ± 4.7 | 0.035^b^ |
|  |  |  |  |
| **Number of medications** |  |  | 0.049^a^ |
| **Preoperative** | 3.4 ± 0.7 | 3.1 ± 1.1 | 0.159^b^ |
| **1 month** | 1.0 ± 1.6 | 0.5 ± 1.3 | 0.335^b^ |
| **3 months** | 1.1 ± 1.6 | 0.6 ± 1.5 | 0.310^b^ |
| **6 months** | 0.9 ± 1.4 | 1.2 ± 1.8 | 0.534^b^ |
| **9 months** | 1.1 ± 1.4 | 1.6 ± 2.0 | 0.282^b^ |
| **12 months** | 1.4 ± 1.6 | 1.8 ± 2.0 | 0.402^b^ |

Data shown in mean ± standard deviation; acBGI = anterior chamber implantation of an Baerveldt glaucoma implant; ppBGI = pars plana implantation of an Baerveldt glaucoma implant; IOP = intraocular pressure

P-value: a, two-way repeated measures ANOVA; b, unpaired *t* test
